# Comparison of Retroperitoneoscopic Versus Transperitoneoscopic Resection of Retroperitoneal Paraganglioma: A Control Study of 74 Cases at a Single Institution

**DOI:** 10.1097/MD.0000000000000538

**Published:** 2015-02-20

**Authors:** Weifeng Xu, Hanzhong Li, Zhigang Ji, Weigang Yan, Yushi Zhang, He Xiao, Xuebin Zhang, Guanghua Liu

**Affiliations:** From the Department of Urology, Peking Union Medical College Hospital, Chinese Academy of Medical Sciences, Beijing, China.

## Abstract

We aimed to compare the safety and patient outcomes of retroperitoneal paraganglioma (PG) following the retroperitoneoscopic and transperitoneoscopic approaches based on large samples.

Seventy-four patients with retroperitoneal PG undergoing laparoscopic resection from June 2004 to September 2013 were retrospectively included. The patients were divided into the retroperitoneal (n = 40) and transperitoneal (n = 34) groups. Demographic and perioperative data, including the operation time, estimated blood loss, incidence of intraoperative hypertension, bowel recovery day, postoperative hospital stay, and systemic inflammatory response syndrome (SIRS) were recorded.

The retroperitoneal group showed a shorter operation time and earlier postoperative exsufflation time compared with the transperitoneal group (84 ± 28.5 minutes vs 115 ± 35.7 minutes and 1.7 ± 0.6 vs 2.3 ± 0.7 day, respectively; both *P* < 0.001). No significant differences in the baseline data were observed between 2 groups. All patients, except for 1 case of open conversion, underwent laparoscopic surgery. There were no patient deaths. Data analysis demonstrated no significant difference in the surgical blood loss, incidence of surgical blood pressure elevation, postoperative hospital stay, or incidence of SIRS between 2 groups.

The operation time for the retroperitoneoscopic resection of retroperitoneal PG is shorter, and gastrointestinal functions improve more quickly compared to the transperitoneoscopic approach. This study may provide a valuable source of clinical information for clinicians in related fields.

## INTRODUCTION

Pheochromocytoma (PCC) is the term used for chromaffin tumor located in the adrenal medulla. Paraganglioma (PG/PGL, previously known as extra-adrenal PCC) is a rare chromaffin cell tumor located extra-adrenally at various sites along the sympathetic and/or parasympathetic chain.^1^ More than 85% of PGs occur below the diaphragm, most commonly seen in the para-aortic region. In addition, PGs can also be found in other locations such as gallbladder, urinary bladder, prostate, spermatic cord, uterus, and duodenum.^2–4^

With large advances in laparoscopic techniques, the laparoscopic resection has become the procedure of choice for most PCC in recent years.^5–7^ Increasing report has proved its safety and feasibility in the treatment of PCC. However, the transperitoneal open approach remains the standard surgical approach for retroperitoneal PGs.^8,9^ Report on the application of laparoscopic techniques in PG treatment remains limited.^7^^,,8,10,11^ Previous study reported 15 cases of PG (6 underwent open surgery, and 9 underwent laparoscopic treatment) and compared them with 62 cases of PCC resected laparoscopically; they concluded that PG could be safely resected laparoscopically despite the long operation time.^12^ Another study reported 161 cases of laparoscopically resected PCC and PG; of which 27 PGs were successfully resected.^10^ However, none of their reports provided the details of the surgical outcomes between the 2 groups. Since most of these studies took the form of a case report and the case number was small, the results were not very persuasive. Laparoscopic resection of PG required thorough investigation in large-scale studies.

The procedure of choice for most PCC is laparoscopic adrenalectomy, either transperitoneally or retroperitoneally; both of which are safe and feasible.^13,14^ The main advantages of the retroperitoneal approach include less interference with the abdominal viscera, a more direct operative route, shortened operative time, fewer postoperative complications, and less pain. However, it has the disadvantages of fewer anatomical landmarks and a smaller operative space when compared with the transperitoneal approach.^13–15^

Recently, several studies have reported the use of transperitoneally or retroperitoneally laparoscopic resection in PG. Kelliher et al proved that the transperitoneoscopic approach was safe and effective for PG located near the aortic bifurcation and inferior vena cava.^16^ Mitchell et al successfully resected PG using the transperitoneal approach facilitated by ultrasound the resection.^17^ Walz et al reported 6 patients undergoing retroperitoneoscopy and 2 undergoing transperitoneoscopy, and their results suggest that the laparoscopic resection of PG is feasible.^8^ However, to the best of our knowledge, no comparative studies on the retroperitoneoscopic and transperitoneoscopic resection of retroperitoneal PG have been conducted.

In this study, the laparoscopic resection of 74 cases of retroperitoneal PG was presented over a 10-year period in a single center. The aim of this study was to evaluate the safety and efficacy of the laparoscopic management of retroperitoneal PG and to compare the outcomes of the retroperitoneal approach with transperitoneal approach. The findings may provide a valuable source of information for clinicians in related fields.

## MATERIALS AND METHODS

### Data

A retrospective analysis of laparoscopic resection for retroperitoneal PG was performed at Peking Union Medical College Hospital from June 2004 to September 2013; cases involving multiple PGs or undergoing other surgery simultaneously were excluded. A total of 74 patients (31 men, 43 women) were included. The patient age ranged from 10 to 75 years old (mean age, 36.9 ± 13.7 years). Six cases were familial (5 MEN-II cases, 1 VHL case). There were 3 recurrent cases (3, 4, and 8 years after previous surgery). The mean tumor size was 4.98 ± 1.38 cm (2.00–8.50 cm).

Fifty-seven patients presented with hypertension, and the remaining 17 patients were incidentally diagnosed with retroperitoneal PG during imaging studies performed for another purpose. All tumors were diagnosed by biochemical findings and localized with the aid of computed tomography, magnetic resonance imaging, or both. ^131^I-meta-iodobenzylguanidine scintigraphy was used in selected cases, particularly in those with congenital disorders.

Retroperitoneal PGs vary in position, which affect their relationships with surrounding anatomical structures. To better demonstrate the distribution of the PGs, we divided the PG distribution into 5 regions: region 1 is on the left side of the aorta ventralis, above the renal pedicle; region 2 is on the left side of the aorta ventralis, under the renal pedicle; region 3 is on the right side of the postcava, above the renal pedicle; region 4 is on the right side of the postcava, under the renal pedicle; and region 5 is between the aorta ventralis and the postcava. Ten tumor cases were located in region 1, 27 cases in region 2, 19 cases in region 3, 10 cases in region 4, and 8 cases in region 5.

This study obtained the approval of the Ethics Committee and Institutional Review Board of Peking Union Medical Hospital. All participants signed an informed consent form. A copy of the written consent is available for review by the Editor of this journal.

### Preoperative Preparation

Preoperatively, all patients received α-adrenergic blockade (phenoxybenzamine), starting with 15 mg/day and gradually increasing to 30 to 90 mg/day. Occasionally, if tachycardia developed, β-adrenergic blockade was added after α-adrenergic blockade. The last dose of phenoxybenzamine was given the morning before surgery. The pharmacological preparation time was 2 to 4 weeks.

### Surgical Approach

Laparoscopic procedures were performed using a retroperitoneal or transperitoneal approach. All surgeries were performed by an operation team led by the same surgeon. The surgeon was experienced in the laparoscopic resection of PCC and had completed hundreds of cases of retroperitoneoscopic and transperitoneoscopic resections. Forty retroperitoneoscopic procedures and 34 transperitoneal approaches were performed, respectively. The surgical approach was based on the anatomical location of the tumor. For tumors above the renal pedicle (regions 1 and 3), the retroperitoneal approach was preferred. For tumors under the renal pedicle (regions 2 and 4), the transperitoneal approach was preferred. For tumors in region 5, the retransperitoneal approach was used. For relapsed cases following open transperitoneal surgery, the retroperitoneal approach was used.

### Surgical Technique

#### Retroperitoneal Approach

The procedure was performed with the patient in the lateral decubitus position. Initially, a 1.5 cm transverse incision was made 2 cm above the iliac crest at the midaxillary line. The retroperitoneal space was reached by blunt and sharp dissection of the abdominal wall. A small cavity was digitally prepared for the subcostal insertion of one 5 mm trocar and one 10 mm trocar in the preaxillary and postaxillary lines, respectively. The retroperitoneal space was maintained with a CO_2_ pressure of 15 mm Hg. A 10 mm, 30° endoscope was introduced into the trocar nearest the iliac crest. The kidney was fully mobilized until the tumor was well visualized. It was sometimes necessary to place a fourth trocar to retract the kidney. The PGs had no consistent blood supply patterns. Major vessels and small branches should be carefully identified and separated by electrocoagulation or clip application. The tumors can then be completely dissected from the surrounding tissue. The incision was enlarged based on the tumor size, and the tumor was removed through a retrieval bag.

#### Transperitoneal Approach

The patient was placed in a lateral decubitus position. The trocar positioning, size, and number depended on the localization of the tumor. Three or 4 trocars were typically required. The first trocar was routinely inserted with an open minilaparotomy above the umbilicus. Pneumoperitoneum was established and maintained at up to 15 mm Hg. One 10 mm trocar and one 5 mm trocar were placed on the lateral border of the rectus muscle (below the umbilicus and subcostally, respectively). A fourth 5 mm port was placed on the midline midway between the xiphoid and umbilicus to provide retraction. The retroperitoneal area was reached by wide mobilization of the mesocolon and mesentery. Thus, the lower abdominal aorta or the lower abdominal vena cava as well as the renal vessels were clearly visualized. Careful dissection was performed to separate the neoplasm from its neighboring large blood vessels and tissues until the tumor was fully removed. The tumor was placed in a retrieval bag and removed.

### Observation Index

Demographic and perioperative data, including the operation time, estimated blood loss, incidence of intraoperative hypertension (defined as a blood pressure measurement >180/90 mm Hg but within normal levels before the anesthesia induction), bowel recovery day, postoperative hospital stay, and SIRS, were recorded and compared. Based on the outcomes, the feasibility of retroperitoneoscopic resection of PGs was assessed, which constituted the primary endpoint of this study.

### Statistical Analysis

All statistical analyses were completed with SPSS 17.0 (SPSS Inc, Chicago, IL). Numerical demographic and perioperative data are expressed as the mean ± SD and were analyzed using Student *t* test. Categorical data, including the male/female ratio, incidence of blood transfusion, incidence of intraoperative hypertension, and the incidence of SIRS, were compared using Pearson χ^2^ test (Fisher exact probability test). P < 0.05 was considered statistically significant.

## RESULTS

No significant differences in the baseline data, including age, gender, body mass index, and tumor volume, were observed between 2 groups (Table 1).

**TABLE 1:**
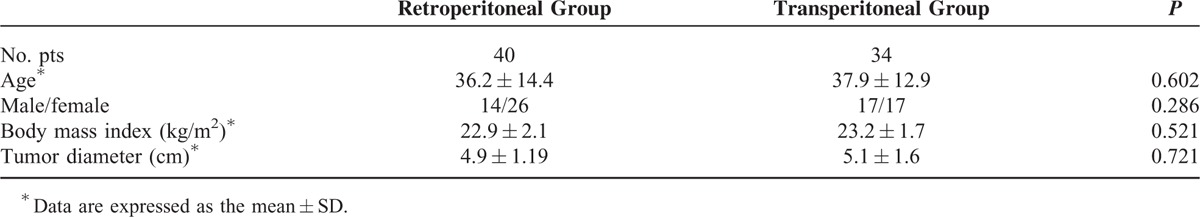
Demographic Data and Tumor Characteristics in the 2 Groups

**FIGURE 1:**
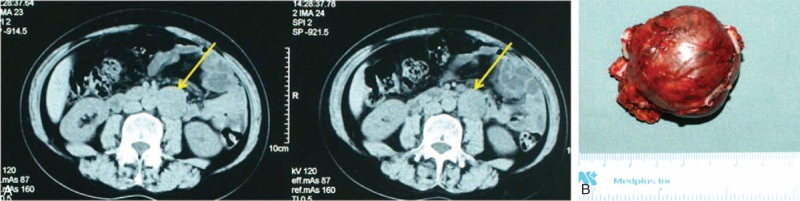
A. Computed tomography (CT) revealed retroperitoneal paraganglioma in the left side. B. General photograph of paraganglioma.

All tumors, except 1, were successfully removed laparoscopically, including 3 recurrent cases. One patient underwent conversion to open surgery due to a dense adhesion of tumor to the vena cava. No significant differences in intraoperative factors, including the amount of blood loss, percentage of blood transfusion, and blood pressure elevation during the operation, were observed between the retroperitoneal and transperitoneal groups (Table 2). The retroperitoneal approach required significantly less operative time than the transperitoneal approach (84 ± 28.5 vs 115 ± 35.7 min, *P* < 0.001). The representative figure of PG was shown in the Figure 1.

**TABLE 2:**
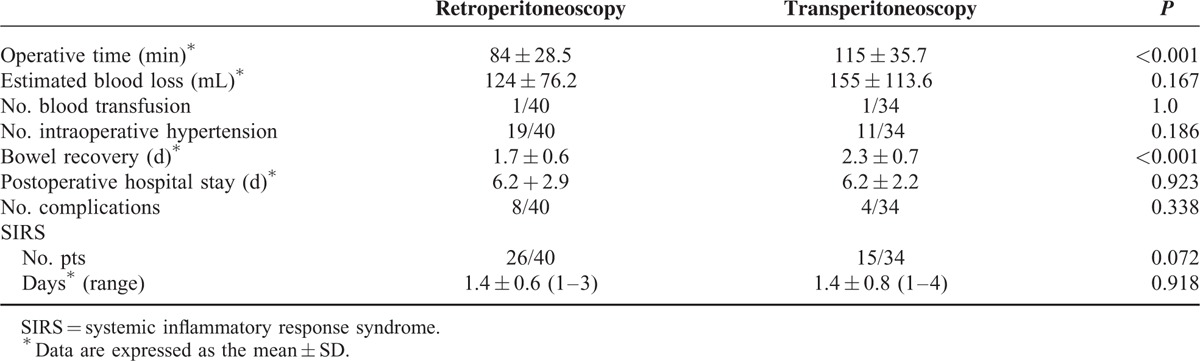
Perioperative Data in the Retroperitoneoscopic and Transperitoneoscopic Surgery Groups

There was no significant difference in the incidence of postoperative SIRS or the length of postoperative hospital stay between 2 groups. In the retroperitoneal group, intestinal function recovered earlier when compared with the transperitoneal group (1.7 ± 0.6 vs 2.3 ± 0.7 day, *P* < 0.001).

None of the included patients died. The intraoperative and postoperative complications in the retroperitoneal group included 1 incision infection, 1 retroperitoneal hematoma, and 6 segmental relaxations of the abdominal wall. In the transperitoneal group, the complications included 1 wound infection, 1 lymphatic leak, and 2 segmental relaxations of the abdominal wall. There were no significant differences in postoperative complications between groups (*P* > 0.05). All cases were managed conservatively without the need for reoperation.

The pathological findings for all resected specimens confirmed the diagnosis of PG. All patients were followed up from 3 to 92 months (mean, 48 months) with CT and catecholamine monitoring. No recurrence or metastasis occurred.

## DISCUSSION

In this study, we compared the outcomes between the retroperitoneal and transperitoneal approaches in the treatment of PG. To the best of our knowledge, this study included the largest number of laparoscopic PG resection cases in a single study. The results showed that although both approaches were feasible for PG, the retroperitoneal approach required less operative time.

For PG, surgery should be carefully performed because of the serious hemodynamic side effects of catecholamines released during operatively. The standard approach to PG is the transperitoneally open surgery.^8,9^ Although the laparoscopic approach has become the standard surgical treatment for adrenal PCCs, its applications in PGs have lagged behind. Since its higher incidence of malignancy, multifocality and the close association with major vascular structures, laparoscopic approach in PGs requires more investigations.^10,18,19^ In the present study, 74 cases of retroperitoneal PG were treated using a laparoscopic approach (either retroperitoneally or transperitoneally). No patient deaths occurred with only 1 patient required conversion to an open procedure. Two patients required blood transfusions. Twelve minor complications occurred, but no reoperation was needed. Our results are consistent with those reported in the previous literature.^8,12,16,17^ These findings indicate that both laparoscopic techniques are safe options for removing PGs for experienced surgeons. Furthermore, it is known that PG has a higher rate of malignancy than adrenal PCC.^10,18,19^ In this study, no relapse or metastasis was observed during follow-up. This might be due to the individual features of the cases, such as the presence of only a single neoplasm and no obvious tissue invasion.

The operation time of retroperitoneal approach is less. In addition, it causes fewer disturbances to the abdominal organs when compared with the transperitoneal approach.^8,17^ Since the retroperitoneal approach provides fewer anatomical landmarks, surgeons must be acquainted with the retroperitoneal anatomy and experienced in retroperitoneal laparoscopic surgery. In addition, the limited retroperitoneal space increases the operative difficulties for large tumors. We chose transperitoneoscopic or retroperitoneoscopic approaches for retroperitoneal PG depending on the site of the neoplasm. Tumors situated caudal to the renal vessels were predominantly excised transperitoneally. Most PGs situated cranial to the renal vessels were removed using the posterior retroperitoneal approach. For interaortocaval tumors, we chose the transperitoneoscopic approach. The transperitoneoscopic approach is associated with some advantages. First, clear anatomical landmarks are visible using this approach, and surgeons can easily identify important structures. Second, with sufficient space, particularly for tumor volumes >6 cm, the transperitoneal approach provides better space for the operation. In addition, using the transperitoneal approach, surgeons can distinguish tumors from the great vessels, including the postcava and aorta ventralis, which may help assess the resectability and avoid injury to the great vessels. In cases of vessel injury, the exposure and control of bleeding could be performed more easily using the transperitoneal approach. Certainly, for transperitoneal approach, it is necessary to dissect adjacent structures, such as the colon, which consumes more surgical time. Furthermore, disturbance of the intestinal tract could be greater. The comparison between 2 groups demonstrated that the operation time for the retroperitoneal approach was shorter than that of the transperitoneal approach and that postoperative exsufflation occurred earlier in the retroperitoneal approach compared with the transperitoneal approach. The advantages of the retroperitoneal approach include a shorter operation time and fewer disturbances of adjacent organs. As PGs vary in origin, size, and localization, the ideal approach depends on the individual situation. In addition, 3 patients suffered from PG relapse after open surgery. The tumors were successfully resected retroperitoneoscopically. This finding suggests that relapsed PG can also be resected laparoscopically and that the retroperitoneal approach can prevent the occurrence of adhesions caused by previous peritoneal open surgery.

This study has some limitations. First, it is a retrospective study and is therefore susceptible to all limitations and biases inherent in a retrospective design. Second, the surgical approach was chosen based on experience rather than random grouping, and therefore, experiential bias might be present. Third, although we divided the distribution of PGs into 5 regions, the positions of the retroperitoneal PGs were not consistent. To overcome these limitations, prospective matched-pair studies are needed in the future.

In conclusion, for experienced surgeons, laparoscopic resection is a safe and efficient strategy for retroperitoneal PG. Because of the various retroperitoneal PG sites, the treatment choice should be based on multiple factors such as the tumor site, volume, and its relationship with adjacent structures. The retroperitoneal approach requires less operation time, and gastrointestinal function may recover earlier.
